# Ericaceous dwarf shrubs in drained forested peatlands: distribution, dynamics, and key factors in a restoration experiment

**DOI:** 10.1093/aobpla/plaf003

**Published:** 2025-01-18

**Authors:** Triin Tekko, Asko Lõhmus

**Affiliations:** Institute of Ecology and Earth Sciences, University of Tartu, J. Liivi 2, Tartu 50409, Estonia; Institute of Ecology and Earth Sciences, University of Tartu, J. Liivi 2, Tartu 50409, Estonia

**Keywords:** afforestation, BACI experiment, ecological restoration, mire vegetation, species coexistence

## Abstract

Ericaceous dwarf shrubs comprise a key component of the vegetation in several types of northern peatlands. Widespread draining of the peatlands is known to favour forest species (such as *Vaccinium myrtillus* and *Vaccinium vitis-idaea*) over mire species (such as *Andromeda polifolia* and *Vaccinium oxycoccos*), but it is unclear to what extent such assemblage shifts should form a target for ecological restoration. In this paper, we analyse the performance of eight co-occurring dwarf shrub species in a large-scale ecological restoration experiment in Scots pine-dominated wetlands that had been drained over 40 years ago in Estonia. We address two related questions: (1) how major ecosystem-change-related factors and within-assemblage interactions affect the 1-m^2^ scale presence of each species in the drained landscape, and (2) to what extent their cover responses to ditch blocking and partial harvest over 6 years reveal a reversal of the drainage-caused succession. We explored those factors and the treatment effects using general linear mixed modelling of the species’ presence and cover. At least four species were responding negatively to drainage, predominantly along with the stand successional stage. However, the results infer that most species were probably enhanced in the early post-drainage phase. The presence of each species was independently enhanced by the presence of other species; the only antagonistic relationship found was between *V. myrtillus* and *V. uliginosum*. Ditch blocking had a clear effect only on *V. oxycoccos*, which increased along with *Sphagnum* moss cover. In several species, we found a temporary decline in some treatments. Overall, the ericaceous shrub cover appeared rather resistant to the fundamental environmental changes investigated and it may serve as a relatively stable functional component both in natural and anthropogenic peatland transitions. In addition to clone longevity, the facilitative mechanisms suggested by co-occurrence patterns may play a role in this and deserve further study.

## Teaser

We used the Before-After-Control-Impact (BACI) approach to study the distribution of dwarf shrubs in drained Scots pine-dominated wetlands and their response to ecological restoration, including combinations of ditch blocking and partial tree harvests. Our findings indicate that the most important factor predicting the presence of individual dwarf shrub species is the presence of other dwarf shrubs. *Vaccinium oxycoccos* was the only species that showed a clear increase in coverage along with the *Sphagnum mosses* in response to the restoration treatments. Overall, the dwarf shrub species proved to be quite resilient to both the historical 40-year drainage and restoration over the course of 6 years.

## Introduction

Ericaceous dwarf shrubs are characteristic plants on acidic soils in northern forests, peatlands, and heathlands where they have a distinct impact on nutrient flows (e.g. Nilsson and Wardle 2005; Ward *et al.* 2009; Fanin *et al.* 2022; Mielke *et al.* 2022). Their ability to access soil nutrients in challenging conditions is related to a mutualistic association with distinct (ericoid) mycorrhizal fungi that can decompose a wide range of organic substrates (Van Geel *et al.* 2020). At the landscape scale, dwarf shrubs play key roles in some fundamental vegetation processes, notably post-fire succession (Nilsson and Wardle 2005; Hart and Chen 2006), forest-heath transitions (Mallik 1995; Fagúndez 2013), woody encroachment in tundra (Vowles and Björk 2019) and bogs (Holmgren *et al.* 2015; but see Hedwall *et al.* 2017), and *Sphagnum* bog replacement with vascular plant cover (Buttler *et al.* 2023). At smaller scales, dwarf shrubs add vertical layer complexity, horizontal patchiness, and key resources (such as berries), which is particularly significant for promoting specialized wildlife in structurally simple ecosystems, such as bogs and pine (*Pinus* spp.) forests (e.g. Wegge and Kastdalen 2008; Mulder *et al.* 2021). The berries of several species are also consumed by humans and berry-picking traditions remain strong in several countries (e.g. Pouta *et al.* 2006; Turtiainen and Nuutinen 2012; Remm *et al.* 2018).

Given such ecological distinctness and importance, the trends and distribution shifts of ericaceous shrubs have come under increasing conservation attention. For example, replacing wildfire disturbances with harvests is generally considered to increase ericaceous shrub cover in forests (Hart and Chen 2006). Yet, a representative dataset across Swedish productive forests revealed their dramatic decrease during the last half a century, which was mostly attributed to forest management and nitrogen deposition effects (Hedwall *et al.* 2019). Another widespread forestry practice with far-reaching consequences on the ground vegetation is the draining of wetlands and wet forests to increase timber production (Paavilainen and Päivänen 1995). The draining initiates a series of cumulative changes in the hydrological and nutrient conditions of soils, vegetation composition and growth, and disturbance dynamics enforced by multiple positive feedbacks (Laine *et al.* 1995; Lõhmus *et al.* 2015). In landscape mosaics, different effects may combine; for example, opposite clear-cutting and peatland drainage effects can re-distribute bilberry (*Vaccinium myrtillus*) in decadal time scales (Lõhmus and Remm 2017). The proximate mechanisms of those effects can be complex; for example, nitrogen addition affects berry production through plant cover, berry formation, and pathogens (Granath and Strengbom 2017).

In this study, we experimentally address this complexity by investigating two-way dynamics in ericaceous shrub assemblages in peatland forests, as subjected to artificial drainage and its removal through ecological restoration. For reversing the drainage-caused processes and conditions, a key activity is to restore hydrology by blocking the ditches, which has been projected to restore original mire vegetation within ca. 20–55 years (Allan *et al.* 2024). However, such ecological reversal to functional mire conditions appears less likely along with the time passed since draining (Vasander *et al.* 2003) and includes much uncertainty and poorly understood spatial heterogeneity (Runnel *et al*. 2023). In this context, the issue of dwarf shrub dynamics emerges twice: as a stabilizing component in both drained and pristine peatlands (Malmer *et al.* 1994; Ward *et al.* 2009) and through its ecological significance to protected wildlife and other conservation targets (Pearce-Higgins and Grant 2006; Boonstra *et al.* 2017; Lõhmus and Remm 2017).

The current knowledge on post-drainage succession and its reversibility in dwarf-shrub assemblages comes from several studies, mostly in Finnish open mires. The research dates back to Sarasto (1961) who distinguished two basic responses of ericaceous shrubs to draining: *V. myrtillus* and *V. vitis-idaea* collectively revealed a gradual two-fold increase, while six mire species (with a total smaller cover than the first group) initially had a double increase and then a similar decline. This generalization soon got further support (Laine *et al.* 1995), although the changes appeared smaller in poor bog sites (Lindholm 1980; Vasander 1982), and, in some systems, mire species showed monotonous post-drainage declines (e.g. Grabovik *et al.* 2021). Interestingly, similar trends have recently been observed also in undrained mires and seem to be accelerating in long-ago drained sites (Ljungqvist 2022). Regarding restoration, the scattered results obtained so far suggest that only a few species of local dwarf shrub assemblages individually respond to the treatments within the up to 15 years monitored. Thus, two studies report a consistent reduction of *Calluna vulgaris*, even below the reference levels (Hancock *et al.* 2018; Johansen 2021). In a 10-year comparative study, in two mire types, *V. myrtillus* reversed its post-drainage increase to a decline in a fen, but little changes were seen in a bog (Haapalehto *et al.* 2011). Also in spruce mires, no clear post-restoration dwarf shrub dynamics appeared, despite a recovery of *Sphagnum* mosses (Maanavilja *et al.* 2014).

We contribute to this knowledge by reporting both drainage and restoration effects on ericaceous shrubs in an experimental system in drained mixotrophic Scots pine (*Pinus sylvestris*) peatlands in Estonia. This system was established to study how drainage has affected critical habitat qualities for a focal bird species, the western capercaillie (*Tetrao urogallus*), and whether the changes can be reversed through ditch blocking and partial cuttings (Lõhmus *et al.* 2017). Dwarf shrubs provide both food resources and shade for the capercaillie (Summers *et al*. 2004; Lõhmus and Remm 2017). The first years of the experiment revealed heterogeneous assemblage changes in the soil and vegetation (Runnel *et al.* 2023), highlighting a need for species-level analyses. Here, we use cover estimates of dwarf shrubs from almost 2000 1-m^2^ quadrats to address this task. First, based on pre-restoration cover estimates and the variation among sites, we explore the role of drainage-related environmental factors (such as adjacency of the ditch, tree composition, and forest succession) in the species’ local distribution. Our specific questions are:

(i) How do soil nutrient status, forest succession, and drainage network contribute to the landscape-scale occurrence of dwarf shrub species?
(ii) How do interactions among dwarf shrub species affect their presence in the landscape?

Secondly, we use the pre- and post-restoration vegetation-cover estimates enabled by our BACI (before-after-control-impact) design to measure the restoration effects, using *Sphagnum* mosses as a sensitive reference group (Maanavilja *et al.* 2014; Buttler *et al.* 2023). Here, the question is:

(iii) How does the dwarf shrub cover respond to ditch blocking and partial harvest over 6 years?

Based on the results, we discuss some future perspectives of drained ecosystems as linked to dwarf shrub cover and its ecological functions.

## Materials and methods

### Study system

**Figure 1.**
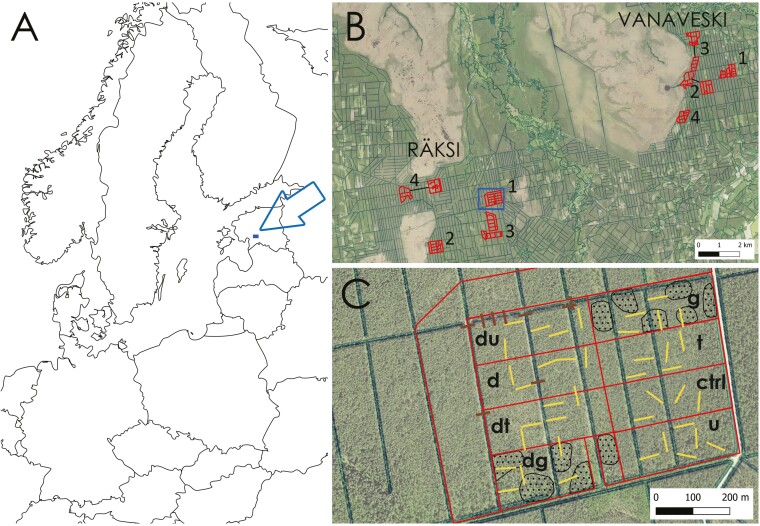
Study design: location in Estonia (A); aerial landscape photograph with ditch and stream network and study clusters (red, numbered 1–4 in both landscapes; (B); treatment design within a cluster (C): ditches were blocked in one half (left; marked with ‘d’) and both halves were randomly subjected to the same three cutting treatments, including a control (ctrl): u—understory removal; t—uniform thinning; g—thinning in gaps (dotted). Plot borders are in red, 50-m transects are in yellow, and dams are in brown. In three clusters, the blocked and drained parts were a few hundred metres apart for logistic reasons (shown as black connection lines in B).

Estonia is a mire-rich, but heavily drained country in the hemiboreal zone of Europe. One-fifth of its land area is peatland, of which 70% has been drained (mostly between the 1950s and 1980s) either for forestry or agriculture (Vasander *et al*. 2003). Our study was conducted in the southwestern part of the country, in the Soomaa region (58° 20′ N; 25° 00′ E; Fig. 1a), which is characterized by large sparsely inhabited forest and mire landscapes. However, the forest areas outside protected bogs are densely ditched (Fig. 1b). Mean annual precipitation in the region is 747 mm and the mean air temperature in July is +17.9°C, and in January –4.0°C (1991–2020 norms; data by Estonian Environmental Agency).

The experimental sites were embedded as clusters in extensive mixotrophic bog-forest areas near large bogs, which can be seen as two landscapes separated by a main road (Fig. 1b). The clusters were situated mostly on hemic histosols (peat thickness ≥50 cm), with some admixture of thinner sapric histosols in the eastern landscape (peat thickness ≥10 cm). The dense ditch network (mean ditch density in forests 8 km/km^2^) in the area was established in 1965–1969, which has expanded the forest area by ca. 30%. Mirroring that, the currently forested experimental areas had on average 70% forest cover in the late 1940s (estimated from topographic maps), the rest being open or semi-open mixotrophic mire. Most of the afforestation has been spontaneous, with (partly failed) Norway spruce (*Picea abies*) planting in one cluster. The drainage has also increased the density and height of existing forest stands. At the start of the experiment (2014), forests in the experimental clusters ranged 50–110 years in age and were dominated (mean: 85%) by Scots pine, with varying shares of downy birch (*Betula pubescens*) and Norway spruce. In addition, half of the forests with a mean age of <100 years contained >100-year-old pines from the pre-drainage sparse stand stage (on average 14% of the overstory; see Lõhmus 2016 for details on stand histories).

Both landscapes contained four experimental clusters, each divided into eight manipulation plots of 2.2–8.4 ha in size (Fig. 1c). In one half of each cluster, the ditches were blocked by building multiple peat dams (83 in total) and where possible, by filling them with the material from the ditch banks, leaving smaller open waterbodies along the former ditch course (details in Vaikre *et al.* 2024). Three randomly assigned plots in each half received distinct tree-cutting treatments, while the fourth was left intact: (i) removal of the undergrowth only (typically spruce, birch, *Salix* spp., and *Frangula alnus*), with the prescription to retain (if present) 20–30 small spruces with branches hanging down to the ground per ha; (ii) understory removal + uniform thinning of ca. 30% of the total volume of the stand; (iii) understory removal + thinning in gaps of 40–100 m in diameter that covered half of the plot. The thinnings took place in the winter of 2014/2015 and the closing of ditches in the winter of 2015/2016. By 2018, the mean canopy cover was reduced from 60% to 44% in thinning-only plots and to 36% when combined with ditch blocking; ditch blocking alone reduced it from 62% to 51% (Runnel *et al.* 2023).

### Field surveys

Field data were collected during the vegetation periods in 2014 (before the treatments), 2016, 2018, and 2022. The cover of each dwarf shrub species was estimated visually in 1-m^2^ quadrats placed after every 10 m along 50-m transects (Fig. 1c). All transects (and quadrats) were at least 3 m away from ditches. The initial transect design was established in the spring of 2014 when five transects per plot were established (plus four additional transects to better capture the most heterogeneous treatment—thinning in gaps; altogether, 324 transects). In the following years, a subset of these were revisited (in 2016, 34 transects in 2 clusters; in 2018, 104 transects in 5 clusters; in 2022, 161 transects in 5 clusters; see Supplementary Table S1). The 2016 survey was focused on bilberry-rich plots (Lõhmus and Remm 2017), while the later surveys attempted to sample treatment variation in the whole setup without prior consideration of the dwarf shrub presence. Importantly, although the transect lines were re-established between (fixed) starting and ending points and relocated with the tape, there was some replacement error of the quadrats; thus, the repeated description of a quadrat could represent a shift up to 1 m from the original quadrat. We assumed that averaging across all quadrats per transect would reduce such possible replacement errors.

The cover of each dwarf shrub species found (*Vaccinium myrtillus*, *V. vitis-*id*aea*, *V. uliginosum*, *V. oxycoccos*, *Rhododendron tomentosum*, *Andromeda polifolia*, *Empetrum nigrum*, and *Calluna vulgaris*) in each quadrat was estimated in percentages at 5% precision (1% precision in the case of cover < 5%). In addition, we measured (i) the percent cover of *Sphagnum* mosses using tape along the transect line at one side of the quadrat; (ii) tree canopy closure (%) visually above each quadrat; and (iii) peat thickness up to 1 m by pushing a sharp stick in the ground (in 2014). The distance to an adjacent ditch was measured by counting steps or digitally by GPS coordinates when the transect was far from the nearest ditch. The data on forest age and tree species composition were obtained from the National Forest Registry (https://register.metsad.ee/).

### Data analysis

We used two linear modelling approaches to address the impacts on ericaceous shrubs by drainage and restoration, respectively. The models were run with R Statistics software (version 4.2.3; R Core Team 2021). The package ‘sjPlot’ (version 2.8.14, Lüdecke *et al.* 2023) was used to visualize the logistic regression models, and ‘emmeans’ (version 1.8.7; Lenth *et al*. 2023) was used to plot the effects of time across treatments.

First, we modelled the presence (1;0) of each species in 2014 across all quadrats (*n* = 1944) using logistic regression (‘lme4’ package, version 1.1-34; Bates *et al.* 2015) in two steps. As a spatial covariance term, a random factor for the ten aggregations of the study plots was included in all the models (Fig. 1b). The ‘performance’ package (version 0.10.4; Lüdecke *et al.* 2021) was used to check the binned residuals and normality of the random effects.

*Step 1* (initial model) tested the contributions of the following continuous factor variables to the presence of the ericaceous shrub species: overstory age and canopy closure (indicators of forest succession); overstory proportion of birch and log_10_-transformed thickness of the peat (indicators of nutrient availability); distance from the ditch, overstory proportion of spruce, and the interaction between the thickness of the peat and the distance from the ditch (indicators of drainage intensity). This set of variables had only weak collinearity (the strongest correlation was between birch proportion and forest age, *r*_*s*_ = −0.121. In this initial step, we eliminated pine proportion from consideration as a potential model variable since it showed a strong correlation with birch proportion, *r*_s_ = −0.84). The independent variables were standardized using the ‘scale’ function in base R (subtracts the mean and divides the result by the standard deviation). The squared terms of overstory age, canopy closure, share of birches, and distance from the ditch were included in the model to explore nonlinear dependencies. The interaction between the log_10_-transformed thickness of the peat and the distance from the ditch was tested to reveal the possible interdependency. If the interaction or the squared terms lacked statistical support (*P* > .05), then they were removed from the model sequentially, beginning with those with the highest *P* values. To the resulting ‘full model’, in *Step 2*, we individually added the presence of *Sphagnum* moss and other dwarf shrubs to explore interspecific competition, facilitation, or potential residual environmental effects not captured in previous steps. For each ‘full model’, we calculated its marginal and conditional *R*^2^ values using the package ‘sjPlot’ (version 2.8.14, Lüdecke *et al.* 2023). We tested the multicollinearity in our ‘full models’ using the ‘vif’ function from the ‘car’ package (version 3.1-2; Fox and Weisberg 2019; the tests revealed that there was no significant multicollinearity in any of our models).

Finally, as a model sensitivity analysis, we ran each ‘full model’ without influential outliers and tested if the same variables remained significant. We determined the outliers using the ‘check_outliers’ function in the ‘performance’ package. In the case of *V. myrtillus* and *V. vitis-idaea*, we reran the models without those outliers (three and two outliers, respectively). For less abundant species, this method identified many outliers and we selected only the most influential ones using the ‘cooks.distance’ function of base R. Only in *V. oxycoccos* and *E. nigrum* removing the outliers rendered a previously significant factor to a level *P* > .05 (details in the ‘Results’ section).

Secondly, we ran linear mixed-effects models (‘lme4’ package, version 1.1-34) for the effects of experimental treatments on the cover of each dwarf shrub species and *Sphagnum* mosses. To reduce quadrat-level variation (including potential placement errors, see above), the dependent variable was the transect-level mean cover (arcsine square-root transformed). The fixed factors were the year, the treatment, and the year × treatment interaction. The random factors were transect ID (to take into account the repeated measurements) and the spatial aggregation term. ANOVA function from the ‘car’ package (version 3.1-2; Fox and Weisberg 2019) was used to test the variables. If the effect was significant in ANOVA, we performed post hoc testing to reveal the specific contrasts using the ‘emmeans package (version 1.8.7; Lenth 2023). The ‘contrast’ function that performs Dunnett’s tests was used to calculate the specific difference from the control treatment for each year, while the ‘pairs’ function that uses the Tukey method was used to calculate the differences between years when the treatment was kept at its mean level. To reveal the differences between the treatment plots at the beginning of the experiment, we used the ‘contrast’ function and calculated the differences from the control in the year 2014. The ‘performance’ package was used to check the plots for residual versus fitted values and quantile–quantile plots.

## Results

### Presence of dwarf shrubs in drained forests

**Figure 2.**
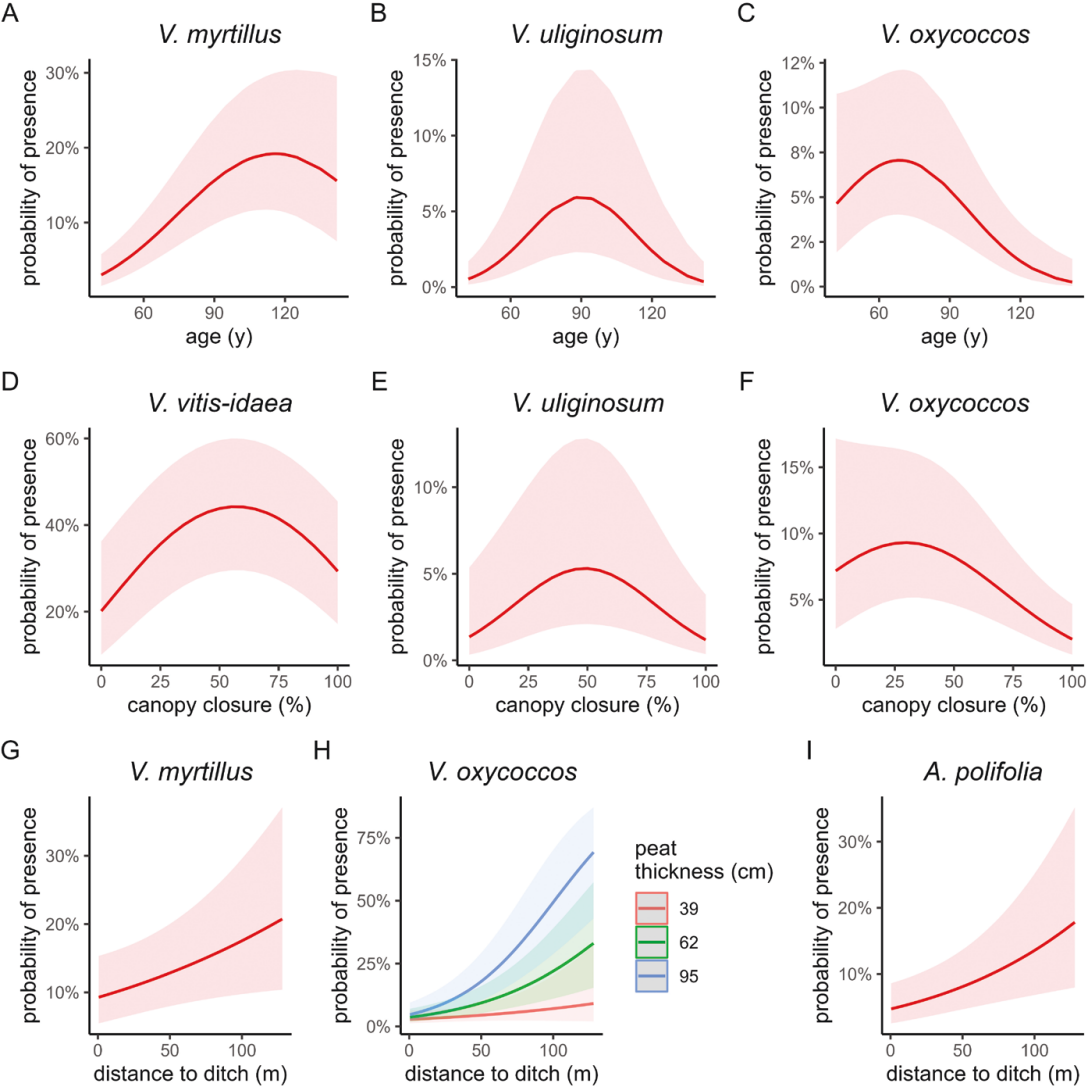
Significant nonlinear effects of the overstory age (A–C) and canopy closure (D–F), and the effect of distance to the nearest ditch (G–I) on the presence of dwarf shrubs in 1-m^2^ squares. A significant interaction of peat thickness and distance to ditch (originally continuous variables) for *V. oxycoccos* has been illustrated as three thickness classes (H).

Tables 1 and 2 summarize the contributions of environmental factors and other species on the presence of ericaceous shrub species in the drained forests, as revealed by the logistic regression models. Major significant effects are illustrated in Fig. 2. There were four main findings:

**Table 1.** General linear models for the presence of four forest- and wooded bog-inhabiting dwarf shrubs in 1-m^2^ ground plots (*n* = 1944) in drained forests.

		Species (no. of presences)							
		*V. myrtillus* (282)		*V. vitis-idaea* (810)		*V. uliginosum* (116)		*R. tomentosum* (95)	
Effect	Factor	Estimate ± SE	*P*	Estimate ± SE	*P*	Estimate ± SE	*P*	Estimate ± SE	*P*
	(Intercept)	−2.034 ± 0.268	<.001	−0.238 ± 0.325	.463	−2.979 ± 0.499	<.001	−4.283 ± 0.537	<.001
N	Birch proportion	−0.159 ± 0.114	.161	0.032 ± 0.098	.740	0.500 ± 0.168	.003	−0.201 ± 0.211	.341
N	(Birch proportion)^2^			−0.197 ± 0.075	.009				
N	Peat thickness (log_10_)	0.125 ± 0.090	.166	−0.185 ± 0.063	.003	−0.057 ± 0.123	.644	0.026 ± 0.154	.867
D	Distance to ditch	0.158 ± 0.074	.032	−0.021 ± 0.054	.703	0.088 ± 0.107	.410	−0.092 ± 0.123	.452
D	Spruce proportion	−0.040 ± 0.102	.690	−0.186 ± 0.068	.006	−0.188 ± 0.134	.162	−1.199 ± 0.316	<.001
S	Overstory age	0.635 ± 0.138	<.001	0.137 ± 0.069	.046	0.647 ± 0.217	.003	−0.272 ± 0.132	.039
S	(Overstory age)^2^	−0.169 ± 0.073	.021			−0.483 ± 0.142	<.001		
S	Canopy closure	0.181 ± 0.074	.014	−0.074 ± 0.059	.214	−0.338 ± 0.127	.008	0.072 ± 0.121	.555
S	(Canopy closure)^2^			−0.174 ± 0.045	<.001	−0.291 ± 0.100	.004		
P	*Sphagnum* moss					0.716 ± 0.235	.002		
P	Other dwarf shrubs (pooled)	0.502 ± 0.164	.002	0.478 ± 0.118	<.001	1.191 ± 0.295	<.001	1.607 ± 0.415	<.001
P	*V. myrtillus*			0.370 ± 0.156	.018	−0.770 ± 0.313	.014		
P	*V. vitis-*id*aea*	0.384 ± 0.155	.013			0.668 ± 0.226	.003	1.148 ± 0.295	<.001
P	*V. uliginosum*	−0.718 ± 0.311	.021	0.687 ± 0.227	.002				
P	*R. tomentosum*			1.035 ± 0.288	<.001	1.390 ± 0.295	<.001		
P	*V. oxycoccos*							0.842 ± 0.342	.014
P	*A. polifolia*					0.577 ± 0.277	0.038	1.275 ± 0.292	<.001
P	*E. nigrum*					1.166 ± 0.472	.014		
P	*C. vulgaris*							1.926 ± 0.600	.001
	Model marginal/conditional *R*^2^	0.108/0.247		0.057/0.270		0.120/0.437		0.221/0.528	

The effects are standardized and broadly categorized as revealing: N*—*soil nutrient status; D*—*drainage intensity; S*—*forest succession; P*—*presence of other plants (as individually added to the model). For the latter, for square terms, and interactions only *P *< .05 effects are shown. A random factor was included in the model to account for spatial covariation. The marginal and conditional *R*^2^ values are calculated for each model not including the presence of other plants.

**Table 2.** General linear models for the presence of four bog- and heath-inhabiting dwarf shrubs in 1-m^2^ ground plots in drained forests.

		Species (no. of presences)							
		*V. oxycoccos* (174)		*A. polifolia* (178)		*E. nigrum* (28)		*C. vulgaris* (18)	
Effect	Factor	Estimate ± SE	*P*	Estimate ± SE	*P*	Estimate ± SE	*P*	Estimate ± SE	*P*
	(Intercept)	−2.611 ± 0.312	<.001	−2.615 ± 0.295	<.001	−8.046 ± 3.916	.040	−5.585 ± 0.854	<.001
N	Birch proportion	0.028 ± 0.133	.836	0.327 ± 0.124	.008	−0.171 ± 0.370	.644	−0.176 ± 0.410	.668
N	Peat thickness (log_10_)	0.605 ± 0.126	<.001	0.324 ± 0.110	.003	0.359 ± 0.311	.248	−0.197 ± 0.260	.447
D	Distance to ditch	0.426 ± 0.097	<.001	0.247 ± 0.085	.004	−0.168 ± 0.236	.477	−0.032 ± 0.249	.898
D	Peat thickness × Distance to ditch	0.217 ± 0.102	.033^*^						
D	Spruce proportion	−0.324 ± 0.116	.005	−0.448 ± 0.122	<.001	0.238 ± 0.263	.366	0.015 ± 0.347	.965
S	Overstory age	−0.193 ± 0.142	.173	−0.088 ± 0.145	.545	0.177 ± 0.435	.684	0.148 ± 0.274	.590
S	(Overstory age)^2^	−0.285 ± 0.112	.011	−0.252 ± 0.104	.015	−0.762 ± 0.358	.033^*^		
S	Canopy closure	−0.462 ± 0.113	<.001	−0.455 ± 0.084	<.001	−0.095 ± 0.202	.637	−0.096 ± 0.241	.691
S	(Canopy closure)^2^	−0.160 ± 0.079	.043						
P	*Sphagnum* moss	1.661 ± 0.215	<.001	0.600 ± 0.201	.003				
P	Other dwarf shrubs (pooled)	0.569 ± 0.202	.005	0.988 ± 0.220	<.001	2.082 ± 1.036	.045	1.498 ± 0.854	.079
P	*V. myrtillus*								
P	*V. vitis-idaea*							1.725 ± 0.861	.045
P	*V. uliginosum*			0.569 ± 0.277	.040	1.155 ± 0.483	.017		
P	*R. tomentosum*	0.871 ± 0.338	.010	1.334 ± 0.295	<.001	1.032 ± 0.540	.056	2.184 ± 0.569	<.001
P	*V. oxycoccos*			1.577 ± 0.210	<.001	1.108 ± 0.455	.015		
P	*A. polifolia*	1.565 ± 0.213	<.001			1.295 ± 0.438	.003		
P	*E. nigrum*	1.142 ± 0.443	.010	1.326 ± 0.424	.002				
	Model marginal/conditional *R*^2^	0.214/0.351		0.160/0.302		0.071/0.847		0.018/0.354	

See Table 1 for other explanations.

^*^ *P* > .05 when removing outliers.

(i) Peatland degradation indicators—*V. vitis-idaea* was the single species with a negative response to peatland features (peat thickness); in addition, the heath-inhabiting species (*E. nigrum*, *C. vulgaris*) had no significant effects attributable to drainage effects (but both were sparse; found in only ca. 1% of plots). In contrast, two species (*V. oxycoccos* and *Andromeda polifolia*) had multiple effects related both to peatland preference and negative drainage impact (ditch distance; proportion of spruce), with a synergistic effect in *V. oxycoccos* (a positive interaction between distance to the ditch and peat thickness; Fig. 2h). Three species only responded to a single peat or drainage indicator: *V. uliginosum* positively to *Sphagnum* presence and *R. tomentosum* negatively to spruce, while the forest species *V. myrtillus* was more frequent farther from ditches (Fig. 2g).

(ii) Stand successional factors were significant for all species except *C. vulgaris*. Three woodland-inhabiting *Vaccinium* species (*V. myrtillus*, *V. vitis-*id*aea*, and *V. uliginosum*) increased along with stand age, but that levelled off or even reversed after ca. 110 and 90 years of age in *V. myrtillus* and *V. uliginosum*, respectively (Fig. 2a and b). In four species, the stand age effects were negative, and in *A. polifolia* and *V. oxycoccus*, these were accompanied by negative effects of canopy closure. In total, five stand-age and three canopy-closure effects were significantly nonlinear (examples in Fig. 2a–f).

(iii) When the peatland and stand successional characteristics were taken into account, all species revealed positive co-occurrence patterns with at least one other ericaceous species (up to four species in *R. tomentosum* and *V. uliginosum*). Also, the pooled effect of all species was positive for each species studied, although it was marginally significant (0.05 < *P* < .1) for *C. vulgaris* (Tables 1 and 2).

(iv) The only negative (antagonistic) co-occurrence pattern was found between *V. myrtillus* and *V. uliginosum*.

### Six-year effects of ecosystem manipulations

We found significant time (year) effects both in *Sphagnum* mosses and all ericaceous species, except *C. vulgaris* (which had a small sample size). Their cover tended to be highest in the last year (2022), that is, 7 years after the thinnings and 6 years after blocking the ditches (Table 3). However, four species (*V. myrtillus*, *V. vitis-idaea*, *A. polifolia, E. nigrum*) experienced temporary declines by 2016 and/or 2018, which were more apparent in the treatment plots (Supplementary Fig. S1).

**Table 3.** Effects from the linear mixed-effects models testing the impact of the year, treatment (plot type), and their interaction (treatment effect over time) on the cover of dwarf shrub species and *Sphagnum* mosses.

Species	*ANOVA P value (Wald)* and significant contrasts (Dunnett’s test for year × Treatment and Treatment, Tukey method for year)					
	Year × treatment		Year		Treatment	
***V. myrtillus***	***0.017***	**2018: dt↓**	***<0.001***	**2014, 2016, 2018 < 2022**	***0.071***	
*V. vitis-idaea*	*0.507*		*<0.001*	2014 > 2016; 2014, 2016, 2018 < 2022	*0.248*	
*V. uliginosum*	*0.502*		*<0.001*	2014, 2016, 2018 < 2022	*<0.001*	ctrl<du, dt
*R. tomentosum*	*0.693*		*0.007*	2016, 2018 < 2022	*0.005*	ctrl<du, dt
*V. oxycoccos*	*0.007*	2022 u, dt↑	*<0.001*	2014, 2016, 2018 < 2022	*0.002*	ctrl<du
*A. polifolia*	*0.261*		*<0.001*	2014 > 2016, 2018; 2022 > 2018	*<0.001*	ctrl<du, dt
*E. nigrum*	*0.406*		*0.014*	2014 > 2018	*0.090*	
*C. vulgaris*	*0.232*		*0.439*		*0.282*	
*Sphagnum*	*0.001*	2022: d, du, dt,↑	*0.003*	2014, 2018 < 2022	*0.085*	

*n* = 623 transect–year combinations. The contrasts of Year × Treatment are shown against the control treatment in each year (the arrows denote directions). Treatment codes: ctrl*—*control, d*—*ditch closure, u*—*understory removal, t*—*uniform thinning, g*—*thinning in gaps.

In turn, *Sphagnum* mosses provided a baseline to assess the much more heterogeneous manipulation effects: sphagna increased by 2022 in the ditch closure plots (Supplementary Fig. S2). The mean increase of ground cover in ditch closure treatments went from 9.3% in 2014 to 11.9% in 2022. *Sphagnum* cover did not respond to cuttings without ditch closure (Table 3). Among ericaceous shrubs, the only species following a similar pattern was *V. oxycoccos*. The only other significant Year × Treatment effect was found in *V. myrtillus*, for which the severest manipulation—a combination of uniform thinning and ditch closure—suppressed its overall increase.

## Discussion

In this study, we conducted an extensive field experiment to examine how dwarf shrubs are distributed in drained forested landscape and how they respond to ecosystem restoration. The main strength of our BACI experimental design was that it enabled us to distinguish the treatment effects from temporal trends across the study system. The distribution of eight dwarf shrub species revealed that the forest species have gained from the drainage of the landscape (indicated by positive associations with the forest age), while the species that prefer wet sites have more complex responses (discussed below). A major factor for the presence of each dwarf shrub species was the presence of other dwarf shrubs, suggesting additional environmental or biotic factors not explicitly captured in our models. The studied species proved quite resistant to the treatments aimed at restoring the pre-drainage mire conditions. Over the six post-restoration years, their cover tended to increase despite temporary declines in some species. *Vaccinium oxycoccos* was the only species that was clearly favoured by the restoration.

### Ericaceous shrub responses to draining

Since ditching in the late 1960s, the studied landscape has been transformed from a semi-open mosaic of bog forests and mires into a more homogeneous landscape covered by early- to mid-successional pine-dominated forests on decayed peat. Along with the developing canopy closure, the ground vegetation has also changed toward a higher abundance of forest species, such as *V. myrtillus* (Lõhmus and Remm 2017). Our results provide insight into how forest succession-related, soil nutrient level-related, and drainage-related factors influence ground vegetation and its stability in drained mixotrophic woodlands.

Perhaps most unexpectedly, once we took into account different indicators of the environmental change, we did not see a direct facilitative effect of draining in any ericaceous species. This contrasts with the continuous increase model suggested for *Vaccinium myrtillus* and *V. vitis-idaea* by Sarasto (1961), and also with an observation that the latter species prefers locations closer to ditches in Estonian mixotrophic mires (Paal *et al.* 2016). We even found an increased frequency of *V. myrtillus* farther from ditches, but, importantly, which was not evident in the same system based on the cover (Lõhmus and Remm 2017). An explanation might be that draining causes its aggregation into larger patches instead of scattered clumps on hummocks (higher small-scale frequency) with little net cover change. A microbiome study also found recently that dwarf-shrub-associated ericoid mycorrhizal dominance is replaced by ectomycorrhiza closer to drainage ditches (Defrenne *et al.* 2023), which suggests a complex response to drainage by the whole symbiotic network.

Since we described the ecosystems nearly half a century after their ditching, the processes inferred from the present vegetation patterns may not fully reveal the whole post-drainage succession and they may also change in the future. Thus, we found no negative effects of *Sphagnum* presence, while such effects are very likely in the wet pristine conditions completely lost by the time of our study. Instead, our models indicated that the main drainage effects on dwarf shrubs at the studied stage are manifested through tree layer succession rather than ground-level hydrology. In terms of the stand age and spruce invasion responses, our results predict further expansion of *V. myrtillus* (likely reaching a stable cover after a century—Fig. 2; see also Hedwall *et al.* 2013), while *V. uliginosum* and *V. vitis-idaea* should turn into a decrease phase after the first half-century increase. In *R. tomentosum*, a similar turn at ca. 25 years post drainage has been reported by other authors (Laine *et al.* 1995), which concurs with our results of its current decline phase (negative effects of stand age and spruce proportion). It is also likely that *E. nigrum* and *C. vulgaris* (now too sparse for conclusive analysis; Table 2) may have had a temporary post-drainage increase phase since both are known to respond negatively to water-logged anoxic conditions (Bannister 1964; Bell and Tallis 1973) presumably common in natural mires. However, in the phase of maturing of post-drainage stands, these two shrub species were probably in decline, as were the most drainage-sensitive species *A. polifolia* and (particularly) *V. oxycoccos*.

We acknowledge that our results remain speculative with regard to the primaeval state of the ecosystem, lost in this system already (Fig. 1b). Nevertheless, a plausible description of the post-drainage succession pattern (cf. Sarasto 1961) is a decades-long co-occurrence of a diverse ericaceous shrub cover, although the contributions of different species vary along the succession. An interesting question to be studied next is the functional role of such ericaceous cover both in vegetation feedbacks (involving soil processes) and as a shade and resource for animal communities. The vegetation feedbacks are probably context dependent, given an array of positive and negative effects described through properties of ericaceous litter, competition versus protection for tree seedlings, facilitating Sphagna by excessive light and heat protection, suppression of ectomycorrhiza, etc. (e.g. Jäderlund *et al.* 1997; Nilsson and Wardle 2005; Holmgren *et al.* 2015; Walker *et al.* 2015; Laine *et al.* 2021; Fanin *et al.* 2022). A key question is whether there might be conditions where dwarf shrubs could stabilize post-drainage tree encroachment and, thus, peatland degradation.

### Species co-occurrence patterns

Strong co-occurrence of ericaceous species at 1-m^2^ scale was a pervasive pattern found in addition to the factors considered. It may refer to either common environmental conditions that were not measured or were measured too coarsely in the drained mixotrophic peatlands, but possible causality deserves attention as well. Instead, spatially heterogeneous, but parallel processes in time are less likely (although possible; e.g. Logofet and Maslov 2019) since, as discussed above, the post-drainage dynamics were not parallel across the set of ericaceous species studied.

Several experimental studies have previously reported similar kinds of ericaceous shrub coexistence for causal reasons not entirely understood (Maillette 1988; Shevtsova *et al.* 1995; Gerdol *et al.* 2000; Duan *et al.* 2022). Among the mechanisms proposed, those most compatible with our results are shared protection against herbivores (Maillette 1988) or litterfall-mediated nutrient effects (Shevtsova *et al.* 1995). In terms of herbivore defense, we observed that the species best known for its insect-repellent chemicals, *R. tomentosum* (Benelli *et al.* 2020), had most frequently positive effects on the presence of other species. In our systems, this relatively tall species was probably not a significant competitor for light due to its scattered distribution. Regarding litter fall, it was notable that the only negative relationship detected was between the only deciduous species—*V. myrtillus* and *V. uliginosum*. These species largely share habitats in Estonia (Lõhmus 1984) and are considered competitors in the Baltic States (Gailīte *et al.* 2020). Perhaps their shorter growing seasons promote competition in the mixotrophic conditions where *V. uliginosum* thrives and becomes a stronger competitor (as described by Gerdol *et al.* 2000). In a different subarctic context, they may even have positive growth relationships, while *V. vitis-idaea* may be a less competitive species (Shevtsova *et al.* 1995).

### Insights from ecosystem restoration

A key feature of our study is that both the drained conditions and post-restoration processes for all ericaceous species have been described in the same ecosystem experiment, which provides a more complete picture of the directions and relative rates of these opposite changes. In the 6-year post-restoration period documented here, we first saw a clear start of an increase in the very low (<10%) *Sphagnum* cover; this constitutes a major vegetation recovery process. The trends in ericaceous shrubs were much less pronounced, including just *V. oxycoccos* recovery in rewetted areas and inhibition of *V. myrtillus* increase when rewetting was combined with thinning. Similar trends have been recorded in a 10-year post-restoration process in a Finnish bog (*Sphagnum* spread) and a fen (*V. myrtillus* retreat; Haapalehto *et al.* 2011). However, thinnings without closing the ditches eventually had a negligible effect on the dwarf shrub cover despite canopy closure being established as a significant factor for several species in the drained stands.

A novel finding was a slight and temporary post-restoration decline in several species. It is known that root damage caused by heavy machinery and covering by the logging slash can temporarily reduce the *V. myrtillus* coverage after thinning (Atlegrim and Sjöberg 1996). Although this could be balanced by improved light conditions, a large-scale study in Finland found an increased cover only since the second decade post thinning (Tonteri *et al.*, 2016). Without ditch blocking, another survey method showed a slight increase of *V. myrtillus* in 2016, 2 years post thinning in our setup (Lõhmus and Remm 2017), while this study showed a decline in the combined thinning + ditch blocking plots in 2018 and a slow recovery by 2022. There was also a reduction of organic nitrogen in the soil by 2018, specifically under the reduced tree canopies (Runnel *et al.* 2023). Thus, we tentatively conclude that the post-restoration declines result from a combination of direct logging disturbance and its indirect soil-mediated effects, which can appear with a delay but are nevertheless transient.

Reasons for the overall increase in the cover of most species over the 8 years remain unclear. Ageing of the tree stands continued throughout the experimental areas, which could affect some general microtopographic, vegetation, and soil developments across all treatments. It is also possible that, because of the close placement of plots, there were some ‘spillover’ effects of the restoration manipulations beyond plot borders that could not be captured through the Year × Treatment terms in our models. It cannot also be excluded that some expansion was due to favourable weather conditions in our study period, given that forest ground vegetation can change notably within short time scales even in undisturbed forests (Økland *et al.*, 1995). The summer of the year 2018 was exceptionally hot and dry (July temperature was 2.5° above average and rainfall 64% below average; Estonian Environmental Agency), possibly favouring dwarf shrubs over graminoids.

Overall, however, the ericaceous shrub cover appeared rather resistant to the fundamental management interventions investigated, which highlights it as a stable functional component both in natural and anthropogenic peatland transitions. A similar conclusion for oligotrophic pine fens has been reached by Laine *et al.* (2011). One biological factor for such stability is apparently the longevity of ericaceous clones (in some species over 100 years) and their predominantly vegetative spread. Another factor may be that their typical microsites on hummocks are less affected by hydrological changes. Although the duration of our study was limited to 8 years, the dwarf shrub cover is unlikely to shift to unprecedented states in the foreseeable future, except perhaps in some wettest locations where we observed expansion of dense reed (*Phragmites australis*). This also indicates that, regarding dwarf shrub-mediated resources and conditions, both drained, restored, and intact peatlands may serve as important habitats for wildlife.

## Supplementary Material

plaf003_suppl_Supplementary_Materials

## Data Availability

The datasets generated and analysed during the current study are available at https://zenodo.org/records/10926331
