# Supplementary material for: Ericaceous dwarf shrubs in drained forested peatlands: distribution, dynamics, and key factors in a restoration experiment
Source: AoB Plants. 2025 Jan 18;17(2):plaf003. doi: 10.1093/aobpla/plaf003 (PMC11829077; doi:10.1093/aobpla/plaf003)
Supplement: plaf003_suppl_Supplementary_Materials [file plaf003_suppl_supplementary_materials.pdf]

Supplementary materials

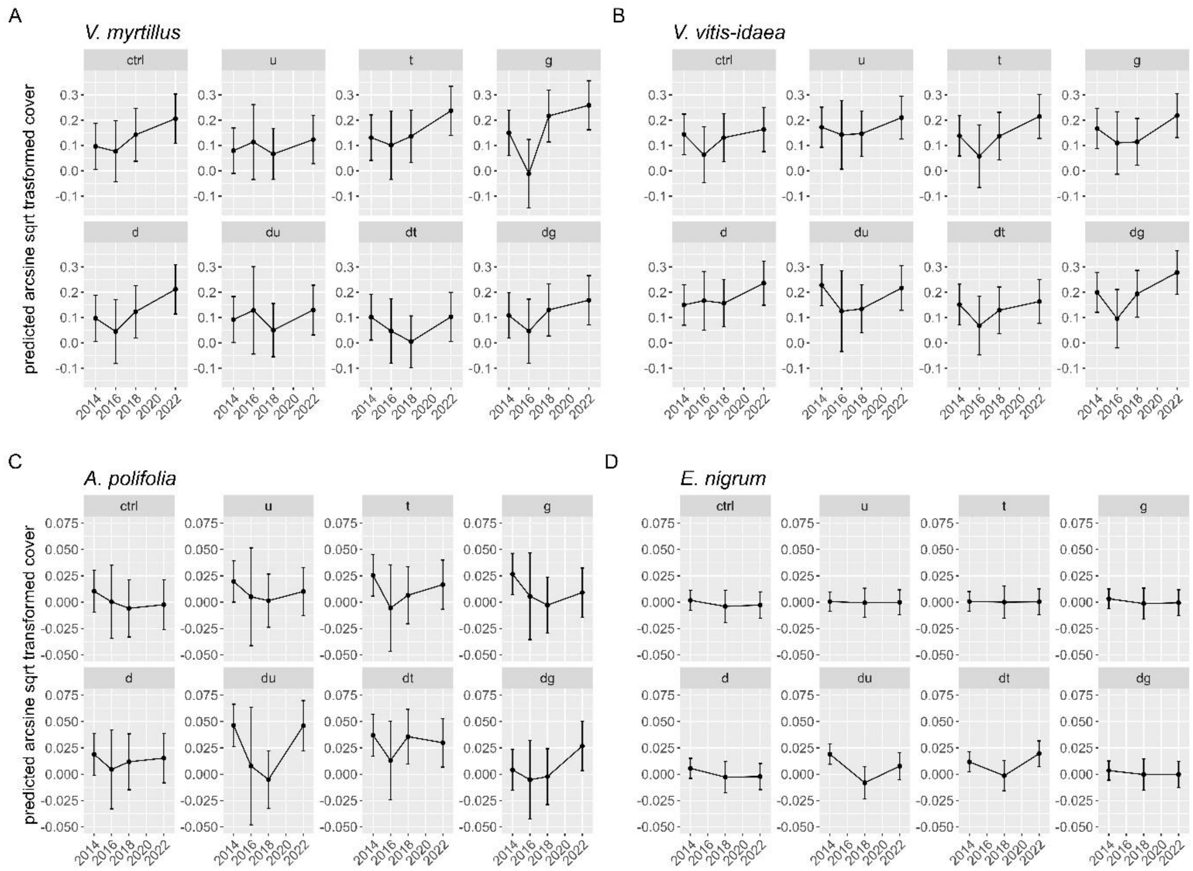

Supplementary Figure 1. Model-predicted cover changes of selected dwarf shrubs in different treatment plots. Depicted are linear predictions of the cover estimates for each studied year, the bars represent 95% confidence intervals. Treatments: ctrl – control; u – understory removal; t – uniform thinning; g – thinning in gaps; d – ditches blocked.

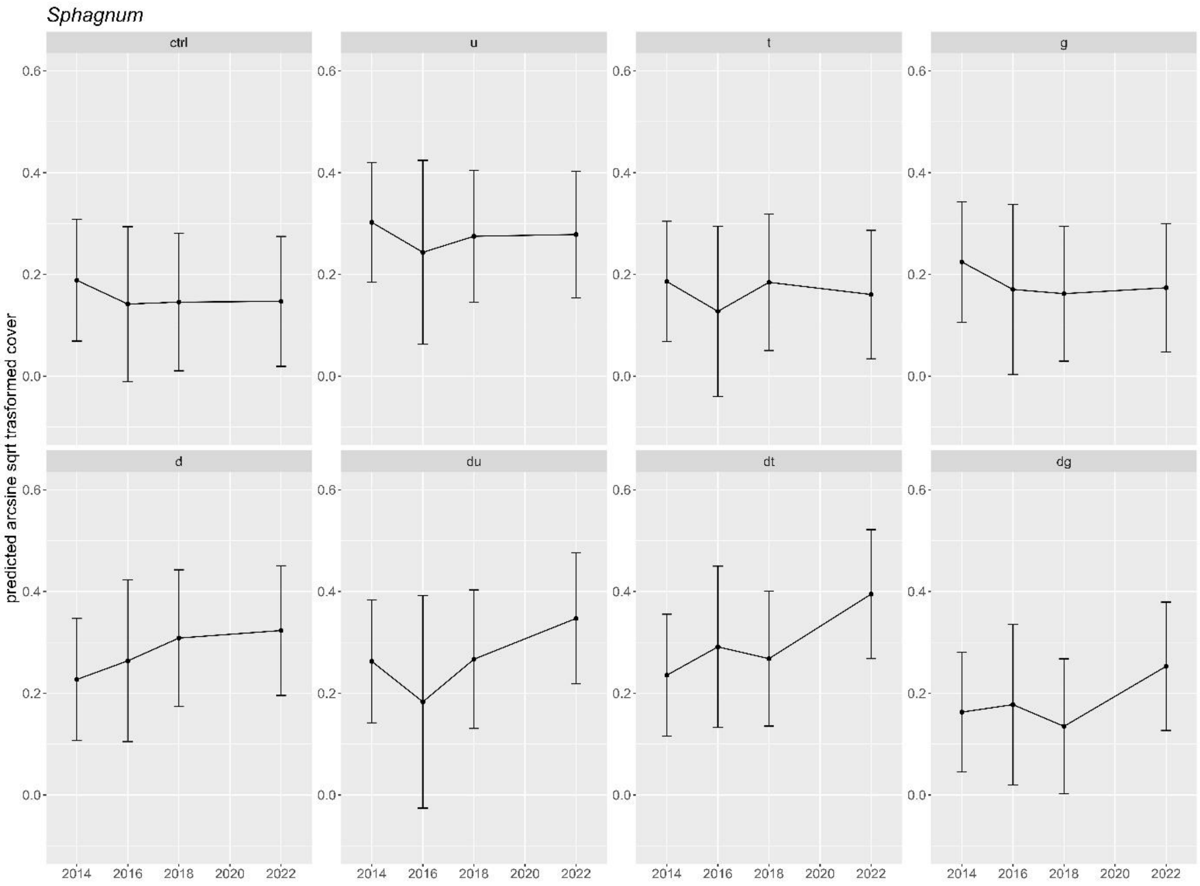

Supplementary Figure 2. Model-predicted cover changes of *Sphagnum* mosses. Depicted are linear predictions of the cover estimates for each studied year, the bars represent 95% confidence intervals. Treatments: ctrl – control; u – understory removal; t – uniform thinning; g – thinning in gaps; d – ditches blocked.

Supplementary Table 1. Counts of transects established in 2014 and revisited in 2016, 2018, and 2022. In 2016 the revisited clusters included Räksi1 and Räksi2, in 2018 and 2022, the revisited clusters included Räksi1, Räksi2, Räksi3, Vanaveski2, Vanaveski4. Since revisiting all the transects was not feasible predominantly due to limited workforce in the follow-up years, we decided to concentrate on revisiting selected clusters, revisiting on average 2-3 transects per cluster in 2018 and 4 transects per cluster in 2022. In 2016, the revisiting was focused on bilberry-rich plots. Treatments: ctrl – control; u – understory removal; t – uniform thinning; g – thinning in gaps; d – ditches blocked.

Treatment	2014	2016	2018	2022
ctrl	40	6	12	19
u	40	3	15	22
t	40	4	12	20
g	40	4	13	20
d	40	5	13	20
du	40	2	12	19
dt	40	5	14	21
dg	44	5	13	20
total	324	34	104	161
